# Overproduction of valuable methoxylated flavones in induced tetraploid plants of *Dracocephalum kotschyi* Boiss

**DOI:** 10.1186/1999-3110-55-22

**Published:** 2014-02-04

**Authors:** Ali Akbar Zahedi, Bahman Hosseini, Mohammad Fattahi, Esmail Dehghan, Hadi Parastar, Hadi Madani

**Affiliations:** 1 Student of Medicinal Plants, Department of Horticulture, Faculty of Agriculture, Urmia University, Urmia, Iran; 2 Department of Horticulture, Faculty of Agriculture, Urmia University, Urmia, Iran; 3 Ph.D. Biotechnology and Plant Breeding, Faculty of Agriculture, Ferdowsi University of Mashhad, Mashhad, Iran; 4 Department of Chemistry, University of Isfahan, Isfahan, Iran

**Keywords:** *Dracocephalum kotschyi* Boiss, Chromosome counting, Flow cytometry, Tetraploidy, HPLC-DAD, Xanthomicrol

## Abstract

**Background:**

Ploidy manipulation is considered an efficient method to increase production potential of medicinally important compounds. *Dracocephalum kotschyi* Boiss. is an endangered medicinal plant of Iran. Various concentrations of colchicine (0.05, 0.10, 0.20, and 0.50% w/v) were applied to shoot apical meristems of *D. kotschyi* seedlings in two and four-leaf stages to induce tetraploidy.

**Results:**

According to the results, 0.5% (w/v) of colchicine can be effective for polyploidy induction in *D. kotschyi*. Putative tetraploids were selected by morphological and microscopic characteristics and their ploidy level was confirmed by flow cytometry analysis and chromosome counting. The chromosome number of original diploid plant was confirmed to be 2*n* = 2*×* = 20 whereas that of the tetraploid plant was 2*n* = 4*×* = 40. Tetraploid and mixoploid plants showed different morphological, physiological and microscopic characteristics from those of diploid counterparts. The total content of flavonoids was increased from 1583.28 in diploids to 1890.07 (μg/g DW) in stable tetraploids.

**Conclusion:**

High-Performance Liquid Chromatography with Diode-Array Detection (HPLC–DAD) confirmed over accumulation of methoxylated hydroxyflavones in solid tetraploid plants of *D. kotschyi*.

**Electronic supplementary material:**

The online version of this article (doi:10.1186/1999-3110-55-22) contains supplementary material, which is available to authorized users.

## Background

*Dracocephalum kotschyi* Boiss, belonging to Labiateae family, is an endemic herbaceous plant and is known as Badrandjboie-Dennaie and Zarrin-Giah (Fattahi et al. 2011; Ghahreman 1987). Recent pharmacological studies have confirmed presence of several methoxylated flavonoids with anti-cancer properties (Jahaniani et al. 2005; Moghaddam et al. 2012) and inhibitory effects on the lectin-induced cellular immune response in this plant (Faham et al. 2008). Its leaf extract has been reported to have antihyperlipidemic (Ebrahim Sajjadi et al. 1998), immunomodulatory (Amirghofran et al. 2000), antinociceptive (Golshani et al. 2004) and cytotoxic (Jahaniani et al. 2005) effects. Aerial parts of *D. kotschyi* plants are sources of valuable flavonoids and essential oils (Ebrahim Sajjadi et al. 1998; Gohari et al. 2003; Monsef-Esfahani et al. 2007; Saeidnia et al. 2007) and its seeds are rich in linolenic, oleic and linoleic acids (Goli et al. 2013).

Flavonoides include over 4000 structurally related compounds existing in nature either as free aglycones or glycosidic conjugates and are generally classified according to their chemical structures into flavones, flavanones, flavanols, flavonols and anthocyanidins (Middleton et al. 2000). This diversity of structural patterns has made flavonoids a rich source of compounds with potential anticancer properties. Recent pharmacological studies suggested some methoxylated flavonoids (Xanthomicrol and Calycopterin) of *D. kotschyi* with anti-cancer properties (Jahaniani et al. 2005; Moghaddam et al. 2012).

Polyploidy has played an important role in genetic and phenotype diversity as well as plant evolution and breeding (Xing et al. 2011). Induction of artificial polyploidy has been considered as a method for increasing production potential of plants secondary metabolites (Dhawan and Lavania 1996; Omidbaigi et al. 2010a). Many polyploid lines of plants were created by application of artificial selective breeding, tissue culture, distant hybridization, physicochemical factors, protoplast culture, and somatic hybridization (Song et al. 2012). However, despite considerable research on artificial polyploidy in plants, very few cases of polyploid medicinal plants have been reported (De Jesus 2003; Dehghan et al. 2012; Lavania and Lavania 2005).

Colchicine (C_22_H_25_NO_6_), originally extracted from *Colchicum autumnale* (autumn crocus, meadow saffron) is a poisonous alkaloid that is extensively used for induction of polyploidy (Ade and Kumar Rai 2010). Colchicine-induced autotetraploid plants has been reported in several medicinal plants including *Tanacetum parthenium* L. (Saharkhiz 2007), *Artemisia annua* L. (Banyai et al. 2010), *Dioscorea zingiberensis* (Zhang et al. 2010), *Dracocephalum moldavica* L. (Omidbaigi et al. 2010b) and *Hyoscyamus muticus* (Dehghan et al. 2012).

Low rate of genetic diversity as well as homogenous population with limited ecological niche in the natural habitat are the main problems in plant breeding (Fattahi 2012). Excessive harvesting of wild plants, limited distribution areas, and a lack of cultivation and domestication are the main reasons that *D. kotschyi* is now listed as an endangered plant (Jalali and Jamzad 1999). Therefore, induction of genetic variation is vital for protection and domestication of this plant.

In this study, we have established a protocol for the induction of tetraploidy in *D. kotschyi* L. for the first time. We also aimed to increase its genetic variation and producing new genetic material for selection of plants with higher production potential of important flavonoid compounds.

## Methods

### Plant material

Seeds of *D. kotschyi* were collected from Chalus, Gachsar, Iran. Seeds were carefully soaked in 98% sulfuric acid for 10 min to remove the external germination inhibitors, according to Fattahi et al. (2011) and then were cultured in plastic pots (15-cm diameter), in a mixture media containing soil, leaf mold and sand (1: 1: 2) and placed in a greenhouse at 25 ± 2°C (days) and 17°C (nights), under a 16/8 h photoperiod at 65% relative humidity.

### Induction of tetraploidy in *D. kotschyi*

In the present study, different concentrations of colchicine (0.0, 0.05, 0.10, 0.20, 0.50% w/v) were applied to shoot apical meristems of two and four-leaf stages plantlets for 48 h using cotton ball method (Shahriari et al. 2009). At the 6^th^ or 8^th^ plant-leaf stages, the plantlets were assessed for the presence of different morphological characteristics from those of diploid control plants including the lengths and widths of stomatal guard cells, plant height and number of leave and side branches.

### Determination of ploidy level

#### Flow cytometry analysis

In order to determine DNA ploidy of the putative tetraploids, flow cytometry analysis (FCM) was conducted by using a Partec, PA, flow cytometer equipped with a mercury lamp (Partec, Germany). Nuclei suspension was prepared by chopping a small piece of fresh leaves (about 0.5 cm^2^) in 400 μl of nuclei extraction buffer (Partec PA, Germany). After filtration through a Partec 30 μm Cell trice disposable filter (Partec) 1600 μl of staining solution containing the dye 4-6-diamino-2-4- phenylindole (DAPI, Partec PA, Germany) was added and a minimum of at least 5000 nuclei per sample were measured and histograms of relative DNA fluorescence were obtained by Mode Fit LT 3.1 software (Dehghan et al. 2012).

#### Chromosome counting

After removing external germination inhibitors of seed coats, as described by Fattahi et al. (2011), seeds were sterilized with 70% ethanol and sodium hypochlorite for 30 s and 20 min respectively, then washed three times by sterile distilled water for 30 min and were placed on wet filter paper in petri dishes and incubated at 25°C until they germinate. Germinated seeds with minimum root length of 2 cm, were pretreated in a saturated solution of 8-hydroxyquinoline 0.02 M at 4°C for 4 h and then at room temperature in dark for 1 h. The samples were then fixed in cold freshly prepared Carnoy’s fixative (ethanol and glacial acetic acid (3: 1)) at room temperature for 3 h and stored in 70% ethanol at 4°C for 12 h. After hydrolyzing by 1 N HCl at 60°C for 10 min, seeds were incubated in Aceto-Iron-Hematoxylin solution at 60°C for 45 min, and squashed on slides in 45% acetic acid-glycerol (9: 1). The chromosomes were observed with the light Olympus microscope (BH2) at 100X under oil immersion objective lens, and the best metaphase views were photographed with digital camera (Canon, Malaysia).

### Evaluation of anatomical and physiological characteristics

#### Stomata characteristics

Five 8 month-old diploid, mixo- and tetraploid plants were randomly selected and stomatal measurements were conducted on them by. The nail varnish technique (Hamill et al. 1992). Microscopic studies were done under the light microscope (Olympus BH2) at 40X magnification. To determine their length and width, stomata on 25 randomly chosen microscopic fields were counted for each leaf. Counts were taken twice per leaf at random locations across the surface in the unit of 0.15 mm^2^.

#### Chlorophyll determinations

The chlorophyll content (a, b, total) of the leaves was evaluated by previously established extraction method (Arnon 1949). In this method extraction was done by acetone and optical density was read at 645 and 663 μm using a spectrophotometer and finally pigments quantity were calculated by using the formula reported by Arnon (1949).

#### Flowering time and fruit set

Flowering time was the time between seed sowing and the beginning of full flowering (80% flowering) and fruit set was the time between seed sowing and the beginning of fruit setting time among plants with different ploidy levels.

### Comparison of selected morphological treats

Ten plants of each ploidy levels including diploids, tetraploids and chimers (mixoploids) were selected for studying leaf number (L. N.), plant height (P. H.), lateral branches number (L. B. N.), stem diameter (mm) (S. D.), inflorescence length (In. L), No. of flower in inflorescence (N. F. In.), flower height (F. H.) (cm), leaf length (L. L.) (cm), leaf width (L. W.) (cm), leaf diameter (L. D.) (mm).

### Extraction of leaf surface flavonoids

Dried leaf material (200 mg) from three individuals of each population was extracted in separate vials containing 10 ml diethyl ether for 24 h as described in the literature (Fattahi et al. 2013; Vieira et al. 2001). In order to prevent the evaporation of diethyl ether, the vials were kept closed and the extraction was performed in a cold room. After 24 h, the extracts were poured into clean vials and leaves were rinsed with another 5 ml diethyl ether, which were added to the initial extracts. The diethyl ether was incubated in an extraction cabinet until complete evaporation of solvent. After adding 10 ml of 80% methanol to remain solid material, the extracts were filtered (0.45 μm pore size) into clean vials. 1.5 ml of prepared solution was transferred into a clean injector auto HPLC vials for automatic injection.

### HPLC–DAD-ESI-MS conditions

Chromatographic separation was performed using a Knauer HPLC system equipped with a Waters Symmetry Shield column (C8, 3.9 × 150 mm) at a flow rate of 1 ml/min. The column oven temperature was set at 30°C. The mobile phase consisted of an isocratic mixture of 60% of A (2% acetic acid in water) and 40% of B (acetonitrile) during 32 min (Fattahi et al. 2013). The DAD was set at 215 nm to provide real time chromatograms, and UV spectra from 190 to 400 nm were recorded for plant component identification. An LC/MSD-TOF (2006) (Agilent Technologies) instrument was used for compound mass detection. Electrospray ionization (ESI-MS) was performed in both positive and negative modes, at a fragmentation voltage of 215 V (positive) and −175 V (negative). Drying gas temperature was 300°C and drying gas (N_2_) flow was 7.01 L/min, with a nebulizer pressure of 15 psi. Capillary voltages were 3.5 kV (negative) and 4 kV (positive).

### Statistical analysis

This study was done as factorial base experiment in completely randomized design with two factors (different concentrations of colchicine and growth stages of treated plantlets) and ten replicates. Data analyses were carried out with the SAS 9.1 for windows software package (Statistical). Means were compared using Tukey’s Honestly Significant Difference (HSD) at the 1% and 5% probability levels.

## Results and discussion

### Determination of ploidy level

#### Flow cytometry analysis

**Figure 1:**
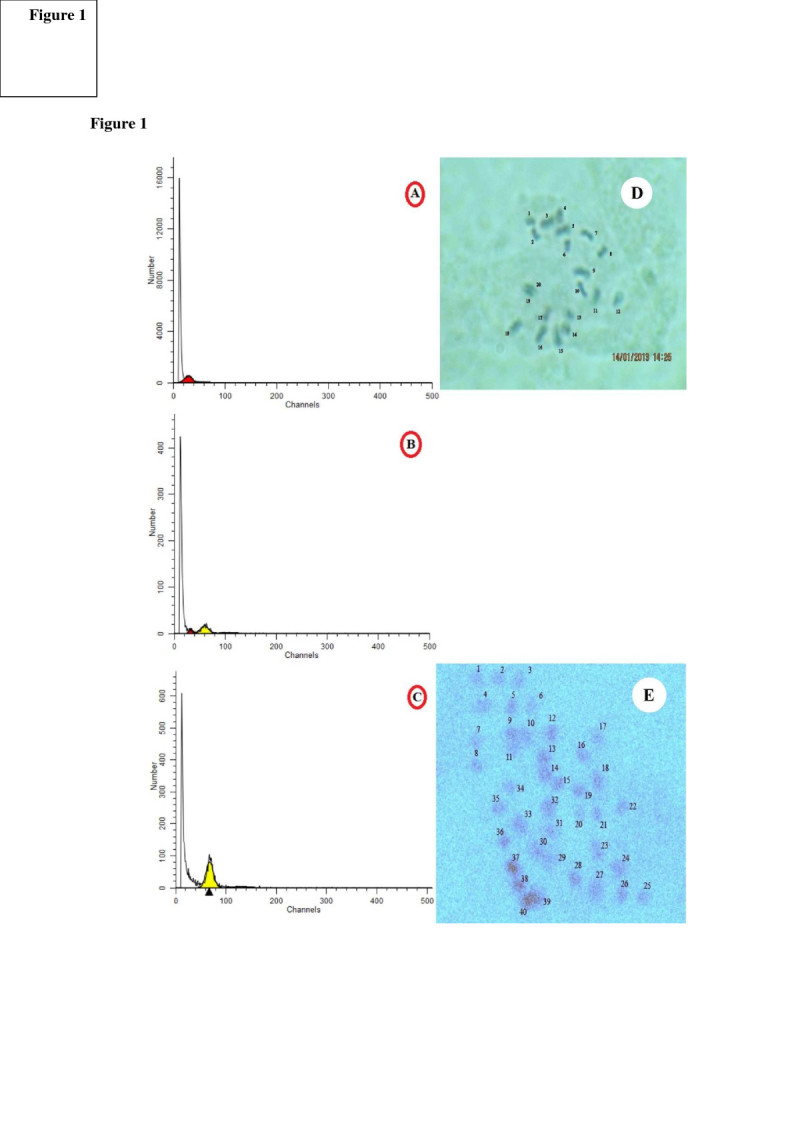
**Flow cytometric histograms [A) 2×, B) 2×-4×, C) 4×], and root tip chromosome number of** ***D. kotschyi*** **in metaphase [D) diploid (2n = 2× = 20) and E) tetraploid (2n = 4× = 40)].**

Flow cytometry analysis of diploid control plants showed a peak at channel 30, related to the G1 of diploid plants (Figure 1a), while the corresponding peak of tetraploids was at about 60 (Figure 1c). Mixoploid plants possess both peaks of 30 and 60 indicating the presence of diploid and tetraploid nuclei (Figure 1b).

#### Chromosome counting

Chromosome counting is the most direct method of ploidy analysis. In line with the flow cytometric data, chromosome counts in tetraploids demonstrated that these plants doubled their diploid chromosome number to 2n = 4× = 40 (Figure 1). To our knowledge there is no report on chromosome counting of *D. kotschyi* and this is the first report on chromosome number of this important medicinal plant.

### Survival rate and growth of colchicine-treated plantlets

The effect of different colchicine concentrations on the survival rate of plants was assessed 30 days after treatment. The survival rate of treated seedlings decreased with increase of colchicine concentration. The highest number of tetraploid plants (12%) and mortality (56%) were recorded when applying 0.5% colchicine to shoot apical meristems of seedlings (Table 1). Only 47% of colchicine-treated plants survived and continued their growth. Colchicine is an antimitotic agent that inhibits the formation of spindle fibers and effectively arrests mitosis at the metaphase stage leading to polyploidy and, as such, has been widely used to induce polyploidy in plant breeding (Abdoli et al. 2013). It has been reported that colchicine results in low growth rate of *Astragalus memberanaceus*, possibility due to physiological damage and a reduced rate of cell division (Chen and Gao 2007).

**Table 1:** **The effect of different concentrations of colchicine treatment on survival rate and ploidy induction of** ***D. kotschyi***

Colchicine (%)	No. of observed plant	Survival rate (%)	Ploidy level (%)		
			Diploid	Mixoploid	Tatraploid
Control	50	100	50 (100)	0	0
0.05	50	76	29 (58)	5 (10)	4 (8)
0.1	50	68	26 (52)	4 (12)	2 (4)
0.2	50	64	25 (50)	6 (8)	3 (6)
0.5	25	44	1 (4)	7 (28)	3 (12)
Total	225	-	131	22	12

The result showed a lower growth rate of tetraploid plants than their diploid counterparts. Colchicine treatments at concentrations of 0.5% and 0.1% (w/v) resulted in induction of 28% and 12% mixoploids, respectively (Table 1).

### The effects of ploidy level on selected anatomical and physiological characteristics

**Figure 2:**
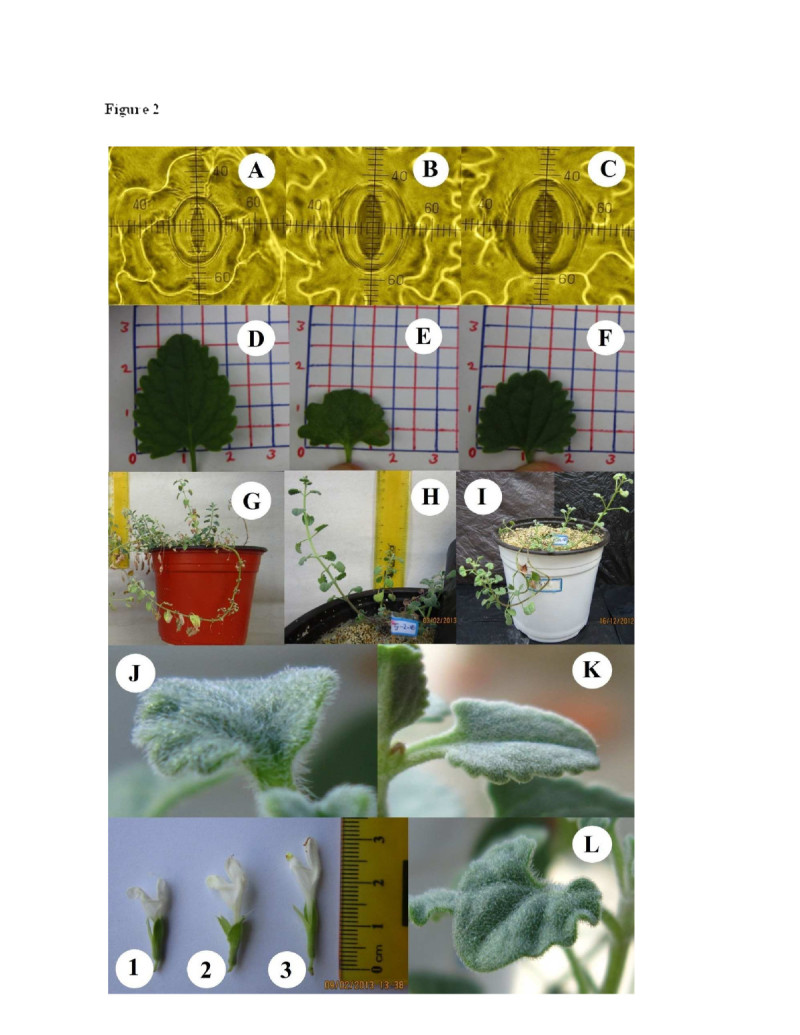
**Comparison of morphological characteristics among diploid, mixoploid and tetraploid plants of** ***D. kotschyi*** **. plant stomata [A) 2×, B) 2×-4×, C) 4×], leaf [D) 2×, E) 2×-4×, F) 4×], Plant morphology [G) 2×, H) 2×-4×, I) 4×], glandular trichome [J) 4×, K) 2×, L) 2×-4×], and plant flower [1) diploid, 2) mixoploid), 3) tetraploid].**

The structural characteristics of *D. kotschyi* were significantly affected by ploidy level. The increase in ploidy level of *D. kotschyi* plants resulted in a significant increase (P <0.01) in stomatal lengths and widths (Figure 2). As shown in Table 2, the number of stomata on abaxial leaf surfaces was also significantly different between diploid and tetraploid plants. Stomatal lengths and widths have been widely used as an indicator of polyploidy (Lin et al. 2011). Generally tetraploid plants tend to have larger stomata than their diploid parents (Gantait et al. 2011; Hodgson et al. 2010; Omidbaigi et al. 2010a; Rêgo et al. 2011; Tang et al. 2010).

**Table 2:** **Some selected anatomical and physiological characteristics of diploid, mixoploid and tetraploid plants of** ***D. kotschyi***

	Stomata			Chlorophyll (mg/g FW)			Trichome density	Planting to flowering time (month)	Fruit set
	Density (no./mm2)	Length (μm)	Width (μm)	a	b	Total			
2×	202.8 ± 28.8 a	20.01 ± 2.42 b	5.63 ± 1.06 b	0.55 ± 0.01 c	0.246 ± 0.03 c	0.80 ± 0.03 c	Low	6	High
2× + 4×	103.7 ± 13.3 b	33.02 ± 3.18 a	11.64 ± 1.77 a	1.01 ± 0.05 b	0.337 ± 0.03 b	1.35 ± 0.07 b	Medium to high	7	Low
4×	68.8 ± 14.0 c	36.42 ± 5.21 a	12.27 ± 1.64 a	1.15 ± 0.05 a	0.658 ± 0.06 a	1.81 ± 0.11 a	High	8	Low

Different letters within the column indicate a highly significant difference of mean (±SD) tested by Tukey’s Studentized Range (HSD) at p ≤ 0.01. The data were analyzed from 10 replications of each treatment.

The chlorophyll (a, b and total) content of tetraploid plants were also significantly changed with ploidy levels (P *< 0.01*) (Table 2). The highest content of chlorophyll a (1.15 mg/g FW) and b (0.65 mg/g FW) were recorded for induced tetraploid plants. Similar results were also reported in colchicine-induced tetraploid plants of *Aloe vera* L. (Li et al. 2002) *Tanacetum parthenium* L. (Majdi et al. 2010) and *Datura stramonium* L. (Amiri et al. 2010).

In this study high and low trichome density were observed on abaxial surface of tetraploid and diploid plants, respectively (Figure 2; Table 2). Similar results were also reported in *Lavandula angustifolia* (Urwin et al. 2007) and *Arachis paraguariensis* (Aina et al. 2012).

The diploid plants generally grew more rapidly than tetraploid plants and began to flower before than tetraploid ones (Figure 2; Table 2). This is in accordance with the results on *Passiflora edulis* Sims. (Rêgo et al. 2011) and *Gerbera jamesonii* Bolus cv. Sciella (Gantait et al. 2011).

Fertility and seed set was lower in induced autotetraploids than their parental diploid plants (Table 2). Similar anatomical and structural changes were also reported in another plants such as *Zinger officinalis* Roscoe (Adaniya and Shirai 2001) and *Dracocephalum moldavica* L. (Omidbaigi et al. 2010b).

### Comparison of selected morphological treats

Leaf length (L. L.) and thickness (L. D.) were significantly affected by induction of tetraploidy (P *< 0.01*), however no significant difference was observed between leaf width (L. W.) of different ploidy levels (Figure 2; Table 3). Tetraploid and mixoploid plants showed a lower L. L. than that of diploid plants (Table 3). Similar results have been reported in other studies (Roy et al. 2001; Viehmannová et al. 2012).

**Table 3:** **Morphological characteristics of diploid, mixoploid and tetraploid plants of** ***D. kotschyi***

Ploidy	Vegetative stage				Flower stage					
	L. N.	P. H.	L. B. N.	S. D. (mm)	In. L. (cm)	N. F. In.	F. H. (cm)	L. L. (cm)	L.W. (cm)	L.D. (mm)
2×	247.8 ± 48.89 a	63.90 ± 10.99 a	55.10 ± 10.24 a	1.376 ± 0.23 b	8.33 ± 3.05 b	13.33 ± 1.52 b	2.33 ± 0.057 b	2.10 ± 0.26 a	1.73 ± 0.24 a	0.302 ± 0.016 c
2× + 4×	91.3 ± 47.47 b	26.20 ± 11.04 b	12.90 ± 9.20 b	1.718 ± 0.18 a	17.50 ± 0.5 a	14.5 ± 0.5 b	3.30 ± 0.435 ab	1.52 ± 0.48 b	1.62 ± 0.38 a	0.412 ± 0.042 b
4×	70.3 ± 36.90 b	20.55 ± 11.09 b	8.50 ± 5.44 b	1.879 ± 0.16 a	17.83 ± 0.76 a	19 ± 1 a	3.33 ± 0.208 a	1.50 ± 0.46 b	1.70 ± 0.33 a	0.481 ± 0.053 a

Different letters within the column indicate a highly significant difference of mean (±SD) tested by Tukey’s Studentized Range (HSD) at p ≤ 0.01. The data were analyzed from 10 replications of each treatment. L. N., Leaf number; P. H., Plant height; L. B. N., lateral branches number; S. D., Stem diameter (mm); In. L. Inflorescence length; N. F. In. No. of Flower in inflorescence; F. H., Flower height (cm); L. L., Leaf length (cm); L. W., Leaf width (cm); L. D., leaf diameter (mm).

Plant growth indices including plant height (P. H.), leaf number (L. N.), lateral branch numbers (L. B. N.) and stem diameter (S. D.) were significantly affected by ploidy level (P < 0.01). While P. H., L. N. and L. B. N. decreased, the S. D. increased in mixoploid and tetraploid plants (Table 3).

Analysis of variance showed a significant increase in corolla length caused by induction of tetraploidy (P *< 0.01*). The highest corolla length (3.33 cm) was recorded in tetraploids while it was 2.33 cm for diploid plants (Figure 2; Table 3). Similar results have been previously reported for *Gerbera jamesonii* Bolus cv. Sciella (Abd El-Naby et al. 2012; Gantait et al. 2011). It seems that higher DNA content and larger cell size in tetraploid plants is associated with the late flowering time (Gantait et al. 2011; Stebbins 1984). There was also a significant difference in Inflorescence length (In. L.) and number of flowers in inflorescence (N. F. In.) between diploid and tetraploid plants (Table 3).

### Identification and quantification of flavonoids by HPLC-DAD-ESI-MS

In order to identification first of all, molecular ion in [M-H]^-^ ions and UV spectra were provided for all compounds (Table 4). According to this table eleven compounds including luteolin-7–O–β-D- glucopyranoside, apigenin 7–O–glucoside (cosmosiin), rosmarinic acid, luteolin 3′–O–β-D-glucuronide, luteolin, apigenin, cirsimaritin, isokaempferide, penduletin, xanthomicrol and calycopterin (Additional file 1) identified by a comparison of the T_R_, UV *λ*_max_, and [M-H]^-^ ions of the *D. kotschyi* peaks with those of known standards (compounds 3, 5, 6, 7, 8, 10 and 11) and quantified by its standard calibration curve and for the others (compounds 1, 2, 4 and 9) identification was done by comparing spectral data reported in previous work (Greenham et al. 2003; Fattahi et al. 2013) and quantified by xanthomicrol calibration curve.

**Table 4:** **Retention time, maximum UV absorption, and molecular weight, flavonoid contents (μg/g DW) in the di-, mix- and tetraploid plants and identification methods of phenolic and flavonoid compounds of** ***D. kotschyi***

Peak	Compound name	RT (min)	UV (nm)	(***m/z)***[M-H]^-^/[M-H]^+^	Diploid	Mixploid	Tetraploid	Identification methods
1	Luteolin-7-O-β-D- glucopyranoside	1.66	205, 255–266, 348	447.09/ -	64.92	116.63	93.82	UV, MS, Ref.
2	Apigenin 7-O-glucoside (cosmosiin)	9.20	233, 269	431.10/ -	32.72	28.59	26.63	UV, MS, Ref.
3	Rosmarinic acid	16.65	234, 290, 329	359.07/ -	938.82	963.96	952.33	UV, MS, Ref., St.
4	Luteolin 3′-O-β-D-glucuronide	17.11	236, 267, 340	461.07/ -	246.48	246.27	252.05	UV, MS, Ref.
5	Luteolin	20.13	232, 267, 344	285.04/ -	20.96	15.78	24.97	UV, MS, Ref., St.
6	Apigenin	22.46	232,268,337	269.04/ -	43.15	42.26	35.86	UV, MS, Ref., St.
7	Cirsimaritin	23.2	276, 232,234	313.07/ -	45.87	42.38	36.48	UV, MS, Ref., St.
8	Isokaempferide	24.68	232, 266, 350	299.05/ -	54.86	31.57	54.35	UV, MS, Ref., St.
9	Penduletin	26.36	234, 272, 340	343.08/345.09	44.69	16.33	80.21	UV, MS, Ref.
10	Xanthomicrol	27.7	232, 282, 333	343.08/345.09	81.75	31.09	140.17	UV, MS, Ref., St.
11	Calycopterin	30.24	233, 278, 337	373.09/375.10	9.06	61.41	193.20	UV, MS, Ref., St.

RT, retention time; Ref or reference, means that compound previously reported by other researchers.

**Figure 3:**
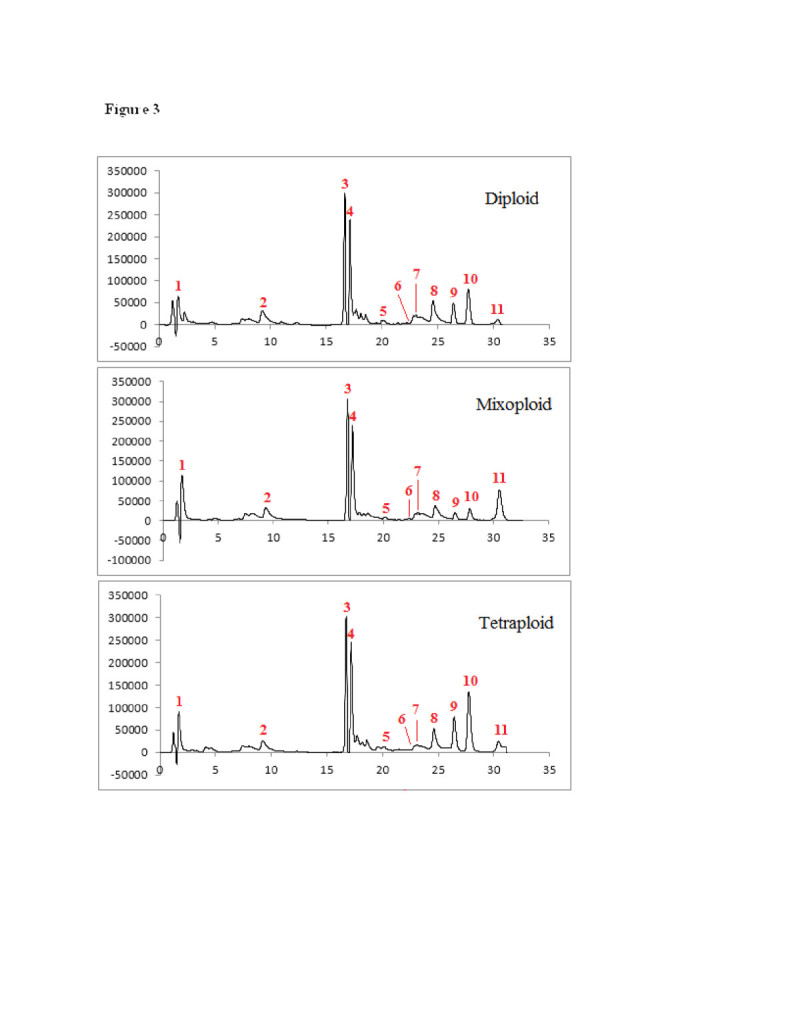
**Chromatograms of HPLC-DAD peaks corresponding to 1) Luteolin-7-** ***O*** **-** ***β*** **-D- glucopyranoside; 2) Apigenin 7-O-glucoside (cosmosiin); 3) Rosmarinic acid; 4) Luteolin 3′-** ***O*** **-.β.-D-glucuronide; 5) Luteolin; 6) Apigenin; 7) Cirsimaritin; 8) Isokaempferide; 9) Penduletin; 10) Xanthomicrol; 11) Calycopterin in: A) Diploid control plant, B) mixoploid, C) tetraploid plant.**

The analytical method applied in this study lending to chromatographic peaks separation shown in Figure 3. In this study quantity of phenolic and flavonoid compounds shown in Table 4. Although the content of hydroxyflavones (apigenin and cosmosiin) was not significantly changed by tetraploidy induction, the results showed that the content of methoxylated flavones (penduletin, xanthomicrol and calycopterin) were increased in tetraploid plants (Table 4). As the ploidy level increased, the percentage of calycopterin, xanthomicrol, penduletin, luteolin, luteolin 3′–*O*–β-D-glucuronide, rosmarinic acid and luteolin-7-*O*-*β*-D- glucopyranoside within the total flavonol pool increased, while the relative concentration of cosmosiin, apigenin, cirsimaritin and isokaempferide decreased. The tetraploid plants could produce about 2 and 21 times higher xanthomicrol and calycopterin compared with their diploid parents, respectively.

Artificial polyploidy generally enhances the concentration of secondary metabolites (Lavania 2013). The mechanism to explain these changes relies on the assumption that chromosome doubling induces an increase in cell size the amount of genetic substance within the nuclei and the nuclear membrane decreases the chromatin is in contact with the nuclear membranes, thereby enhancing gene activity and photosynthetic rate on a per cell basis (Lavania and Lavania 2005; Levin 2002). It may be that with more chromatin coming into contact with the nuclear membrane due to the lower ratio of nuclear membrane to gene dosage after polyploidization, gene activity is elevated (Levin 2002). Changing the metabolic profile in autopolyploid plants by a simple duplication of the basic genome was interpreted as a cause of an alteration in the mechanism(s) which regulates the biosynthesis of individual compounds (Dehghan et al. 2012). Lipophilic flavonoid aglycones have a limited distribution in plants compared to their water-soluble glycosides. As they usually accumulate on the leaf surface, and are found in glandular trichomes or are extruded through the cuticle, they are known as surface or external flavonoids. Aglycone flavonoids, especially in the highly methylated form, accumulate in the Lamiaceae family (Tomás-Barberán and Wollenweber 1990). Therefore another possible reason for flavonoid methoxy enhancement can be because of high glandular trichomes stracture in tetraploid plants. To our knowledge there is only a few reports related to the effects of ploidy level on accumulation of medicinally important flavonoids. Griesbach and Kamo (1996) reported increasing of the major flavonols (quercetin-3-sophoroside) and decreasing of the minor flavonols (quercetin-3,7-diglucoside) in colchicine treated *Petunia* ‘Mitchell’. In another study Abdoli et al. (2013) reported a 71% and 45% increase in cholorgenic acid content and cichoric acid compounds of tetraploid leaves of *Echinacea purpurea* L., respectively. Similar results were also reported for different secondary compounds including apigenin (Švehlíková and Repčák 2000), artemisinin (Banyai et al. 2010), Phenylpropanoid content (Caruso et al. 2011) and scopolamine (Dehghan et al. 2012).

## Conclusion

This study is the first report on induction and establishment of autotetraploid plants of *D. kotschyi*. The highest applied concentration (0.5% v/w) of colchicine, results in the induction of highest number of stable tetraploid plants. Induction of tetraploidy significantly affected different morphological, microscopic, physiological and biochemical characteristics of *D. kotschyi*. These changes suggested ploidy manipulation as a rapid and effective method for enhancing genetic diversity and metabolite production of *D. kotschyi*.

## Electronic supplementary material

Additional file 1:**Chemical structure of compounds in** ***Dracocephalum kotschyi*** **L. corresponding to 1) Luteolin-7-** ***O*** **-** ***β*** **-D- glucopyranoside; 2) Apigenin 7-O-glucoside (cosmosiin); 3) Rosmarinic acid; 4) Luteolin 3′-** ***O*** **-.β.-D-glucuronide; 5) Luteolin; 6) Apigenin; 7) Cirsimaritin; 8) Isokaempferide; 9) Penduletin; 10) Xanthomicrol; 11) Calycopterin.**(DOC 1 MB)

Below are the links to the authors’ original submitted files for images.

Authors’ original file for figure 1

Authors’ original file for figure 2

Authors’ original file for figure 3

## Abbreviations

HPLC–DAD: High-performance liquid chromatography with diode-array detection
FCM: Flow cytometry
LN: Leaf number
PH: Plant height
LBN: Lateral branches number
SD: Stem diameter
InL: Inflorescence length
NFIn: Number of flower in inflorescence
FH: Flower height
LL: Leaf length
LW: Leaf width
LD: Leaf diameter.

## References

[CR1] Abd El-Naby ZM, Mohamed NA, Radwan KH, El-Khishin DA (2012). Colchicine induction of polyploidy in Egyptian clover genotypes. J Am Sci.

[CR2] Abdoli M, Moieni A, Badi HN (2013). Morphological, physiological, cytological and phytochemical studies in diploid and colchicine-induced tetraploid plants of *Echinacea purpurea* L. Acta Physiol Plant.

[CR3] Adaniya S, Shirai D (2001). *In vitro* induction of tetraploid ginger (*Zingiber officinale* Roscoe) and its pollen fertility and germinability. Sci Hortic.

[CR4] Ade R, Kumar Rai M (2010). Review: colchicine, current advances and future prospects. Nusant Biosci.

[CR5] Aina O, Quesenberry K, Gallo M (2012). *In vitro* induction of tetraploids in *Arachis paraguariensis*. Plant Cell Tiss Organ Cult.

[CR6] Amirghofran Z, Azadbakht M, Karimi MH (2000). Evaluation of the immunomodulatory effects of five herbal plants. J ethnopharmacol.

[CR7] Amiri S, Kazemitabaar S, Ranjbar G, Azadbakht M (2010). The effect of trifluralin and colchicine treatments on morphological characteristics of jimsonweed (*Datura Stramonium* L.). Trakia J Sci.

[CR8] Arnon DI (1949). Copper enzymes in isolated chloroplasts. polyphenoloxidase in *Beta vulgaris*. Plant physiol.

[CR9] Banyai W, Sangthong R, Karaket N, Inthima P, Mii M, Supaibulwatana K (2010). Overproduction of artemisinin in tetraploid *Artemisia annua* L. Plant biotechnol.

[CR10] Caruso I, Lepore L, De Tommasi N, Dal Piaz F, Frusciante L, Aversano R, Garramone R, Carputo D (2011). Secondary metabolite profile in induced tetraploids of wild *Solanum commersonii* Dun. Chem Biodivers.

[CR11] Chen L-L, Gao S-L (2007). *In vitro* tetraploid induction and generation of tetraploids from mixoploids in *Astragalus membranaceus*. Sci Hortic.

[CR12] De Jesus L (2003). Effects of artificial polyploidy in transformed roots of Artemisia annua L.

[CR13] Dehghan E, Häkkinen ST, Oksman-Caldentey K-M, Ahmadi FS (2012). Production of tropane alkaloids in diploid and tetraploid plants and *in vitro* hairy root cultures of Egyptian henbane (*Hyoscyamus muticus* L.). Plant Cell Tiss Organ Cult.

[CR14] Dhawan O, Lavania U (1996). Enhancing the productivity of secondary metabolites via induced polyploidy: a review. Euphytica.

[CR15] Ebrahim Sajjadi S, Movahedian Atar A, Yektaian A (1998). Antihyperlipidemic effect of hydroalcoholic extract, and polyphenolic fraction from *Dracocephalum kotschyi* Boiss. Pharm Acta Helv.

[CR16] Faham N, Javidnia K, Bahmani M, Amirghofran Z (2008). Calycopterin, an immunoinhibitory compound from the extract of *Dracocephalum kotschyi*. Phytother Res.

[CR17] Fattahi M (2012). Evaluation of morphological, phytochemical diversity and hairy root production in Dracocephalum kotschyi Boiss.

[CR18] Fattahi M, Nazeri V, Sefidkon F, Zamani Z, Palazon J (2011). The effect of pre-sowing treatments and light on seed germination of *Dracocephalum kotschyi* Boiss: An endangered medicinal plant in Iran. Hort Environ Biotechnol.

[CR19] Fattahi M, Nazeri V, Torras-Claveria L, Sefidkon F, Cusido RM, Zamani Z, Palazon J (2013). Identification and quantification of leaf surface flavonoids in wild-growing populations of *Dracocephalum kotschyi* by LC–DAD–ESI-MS. Food Chem.

[CR20] Gantait S, Mandal N, Bhattacharyya S, Das PK (2011). Induction and identification of tetraploids using *in vitro* colchicine treatment of *Gerbera jamesonii* Bolus cv. Sciella. Plant Cell Tiss Organ Cult.

[CR21] Ghahreman A (1987). Flore de iranica en couleur naturelle, faculty of science.

[CR22] Gohari AR, Saeidnia S, Matsuo K, Uchiyama N, Yagura T, Ito M, Kiuchi F, Honda G (2003). Flavonoid constituents of *Dracocephalum kotschyi* growing in Iran and their trypanocidal activity. Nat Med.

[CR23] Goli SAH, Sahafi SM, Rashidi B, Rahimmalek M (2013). Novel oilseed of *Dracocephalum kotschyi* with high n-3 to n-6 polyunsaturated fatty acid ratio. Ind Crop Prod.

[CR24] Golshani S, Karamkhani F, Monsef-Esfehani HR, Abdollahi M (2004). Antinociceptive effects of the essential oil of *Dracocephalum kotschyi* in the mouse writhing test. J Pharm Pharm Sci.

[CR25] Greenham J, Harborne JB, Williams CA (2003). Identification of lipophilic flavones and flavonols by comparative HPLC, TLC and UV spectral analysis. Phytochem Anal.

[CR26] Griesbach R, Kamo K (1996). The effect of induced polyploidy on the flavonols of *Petunia* ‘Mitchell’. Phytochem.

[CR27] Hamill S, Smith M, Dodd W (1992). *In vitro* induction of banana autotetraploids by colchicine treatment of micropropagated diploids. Aust J Bot.

[CR28] Hodgson J, Sharafi M, Jalili A, Díaz S, Montserrat-Martí G, Palmer C, Cerabolini B, Pierce S, Hamzehee B, Asri Y (2010). Stomatal vs. genome size in angiosperms: the somatic tail wagging the genomic dog?. Ann Bot.

[CR29] Jahaniani F, Ebrahimi SA, Rahbar-Roshandel N, Mahmoudian M (2005). Xanthomicrol is the main cytotoxic component of *Dracocephalum kotschyi* and a potential anti-cancer agent. Phytochem.

[CR30] Jalali A, Jamzad Z (1999). Red data book of Iran: research institute of forests and rangelands Iran Tehran.

[CR31] Lavania UC (2013). Polyploidy, body size, and opportunities for genetic enhancement and fixation of heterozygosity in plants. Nucleus.

[CR32] Lavania U, Lavania U (2005). Genomic and ploidy manipulation for enhanced production of phyto-pharmaceuticals. Plant Genet Resour.

[CR33] Levin DA (2002). The role of chromosomal change in plant evolution.

[CR34] Li W, Sixiang Z, Zhijian G (2002). *In vitro* culture of tetraploids of *Aloe vera* L. Acta Hort Sin.

[CR35] Lin X, Zhou Y, Zhang J, Lu X, Zhang F, Shen Q, Wu S, Chen Y, Wang T, Tang K (2011). Enhancement of artemisinin content in tetraploid *Artemisia annua* plants by modulating the expression of genes in artemisinin biosynthetic pathway. Biotechnol and Appl Biochem.

[CR36] Majdi M, Karimzadeh G, Malboobi MA, Omidbaigi R, Mirzaghaderi G (2010). Induction of tetraploidy to feverfew (*Tanacetum parthenium* Schulz-Bip.): morphological, physiological, cytological, and phytochemical changes. HortSci.

[CR37] Middleton E, Kandaswami C, Theoharides TC (2000). The effects of plant flavonoids on mammalian cells: implications for inflammation, heart disease, and cancer. Pharmacol rev.

[CR38] Moghaddam G, Ebrahimi SA, Rahbar‒Roshandel N, Foroumadi A (2012). Antiproliferative activity of flavonoids: influence of the sequential methoxylation state of the flavonoid structure. Phytother Res.

[CR39] Monsef-Esfahani H, Karamkhani F, Nickavar B, Abdi K, Faramarzi M (2007). The volatile constituents of *Dracocephalum kotschyi* oils. Chem Nat Compd.

[CR40] Omidbaigi R, Mirzaee M, Hassani M, Moghadam M (2010). Induction and identification of polyploidy in basil (*Ocimum basilicum* L.) medicinal plant by colchicine treatment. Int J Plant Prod.

[CR41] Omidbaigi R, Yavari S, Hassani ME, Yavari S (2010). Induction of autotetraploidy in dragonhead (*Dracocephalum moldavica* L.) by colchicine treatment. J Fruit Ornam Plant Res.

[CR42] Rêgo M, Rêgo E, Bruckner C, Finger F, Otoni W (2011). *In vitro* induction of autotetraploids from diploid yellow passion fruit mediated by colchicine and oryzalin. Plant Cell Tiss Organ Cult.

[CR43] Roy A, Leggett G, Koutoulis A (2001). *In vitro* tetraploid induction and generation of tetraploids from mixoploids in hop (*Humulus lupulus* L.). Plant Cell Rep.

[CR44] Saeidnia S, Gohari A, Hadjiakhoondi A, Shafiee A (2007). Bioactive compounds of the volatile oil of *Dracocephalum kotschyi*: Zeitschrift für Naturforschung C, A. J biosci.

[CR45] Saharkhiz MJ (2007). The effects of some environmental factors and ploidy level on morphological and physiological characteristics of feverfew (Tanacetum parthenium L.) medicinal ornamental plant.

[CR46] Shahriari F, Dehghan E, Farsi M (2009). Tetraploid induction of *Hyoscyamus muticus* L. using colchicine treatment. Proceedings of the 14th Austalasian plant breeding, 11th SABRAO conference.

[CR47] Song C, Liu S, Xiao J, He W, Zhou Y, Qin Q, Zhang C, Liu Y (2012). Polyploid organisms. Sci China Life Sci.

[CR48] Stebbins G (1984). Polyploidy and the distribution of the arctic-alpine flora: new evidence and a new approach. Bot Helv.

[CR49] Švehlíková V, Repčák M (2000). Variation of apigenin quantity in diploid and tetraploid *Chamomilla recutita* (L.) Rauschert. Plant Biol.

[CR50] Tang Z-Q, Chen D-L, Song Z-J, He Y-C, Cai D-T (2010). *In vitro* induction and identification of tetraploid plants of *Paulownia tomentosa*. Plant Cell Tiss Organ Cult.

[CR51] Tomás-Barberán FA, Wollenweber E (1990). Flavonoid aglycones from the leaf surfaces of some Labiatae species. Plant Syst Evol.

[CR52] Urwin NA, Horsnell J, Moon T (2007). Generation and characterisation of colchicine-induced autotetraploid *Lavandula angustifolia*. Euphytica.

[CR53] Viehmannová I, Trávníčková M, Špatenková E, Černá M, Trávníček P (2012). Induced polyploidization and its influence on yield, morphological, and qualitative characteristics of microtubers in *Ullucus tuberosus*. Plant Cell Tiss Organ Cult.

[CR54] Vieira RF, Grayer RJ, Paton A, Simon JE (2001). Genetic diversity of *Ocimum gratissimum* L. based on volatile oil constituents, flavonoids and RAPD markers. Biochem Syst Ecol.

[CR55] Xing S-H, Guo X-B, Wang Q, Pan Q-F, Tian Y-S, Liu P, Zhao J-Y, Wang G-F, Sun X-F, Tang K-X (2011). Induction and flow cytometry identification of tetraploids from seed-derived explants through colchicine treatments in *Catharanthus roseus* (L.) G. Don. J Biomed Biotechnol 1–10.

[CR56] Zhang X-Y, Hu C-G, Yao J-L (2010). Tetraploidization of diploid *Dioscorea* results in activation of the antioxidant defense system and increased heat tolerance. J plant physiol.

