# Supplementary material for: Overproduction of valuable methoxylated flavones in induced tetraploid plants of Dracocephalum kotschyi Boiss
Source: Bot Stud. 2014 Feb 4;55:22. doi: 10.1186/1999-3110-55-22 (PMC5430325; doi:10.1186/1999-3110-55-22)
Supplement: Supplementary file 1 — Additional file 1:Chemical structure of compounds in Dracocephalum kotschyi L. corresponding to 1) Luteolin-7- O - β -D- glucopyranoside; 2) Apigenin 7-O-glucoside (cosmosiin); 3) Rosmarinic acid; 4) Luteolin 3′- O -.β.-D-glucuronide; 5) Luteolin; 6) Apigenin; 7) Cirsimaritin; 8) Isokaempferide; 9) Penduletin; 10) Xanthomicrol; 11) Calycopterin.(DOC 1 MB) [file 40529_2013_75_MOESM1_ESM.doc]

Chemical structure:

**Luteolin-7-O-β-D- glucopyranoside (1)**


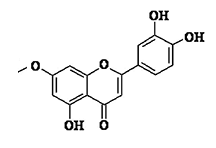


**Glc**

…………………………………………………………………………………………….

**Apigenin 7-O-glucoside (cosmosiin) (2)**


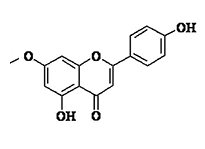


**Glc**

…………………………………………………………………………………………........

**Rosmarinic acid (3)**

**
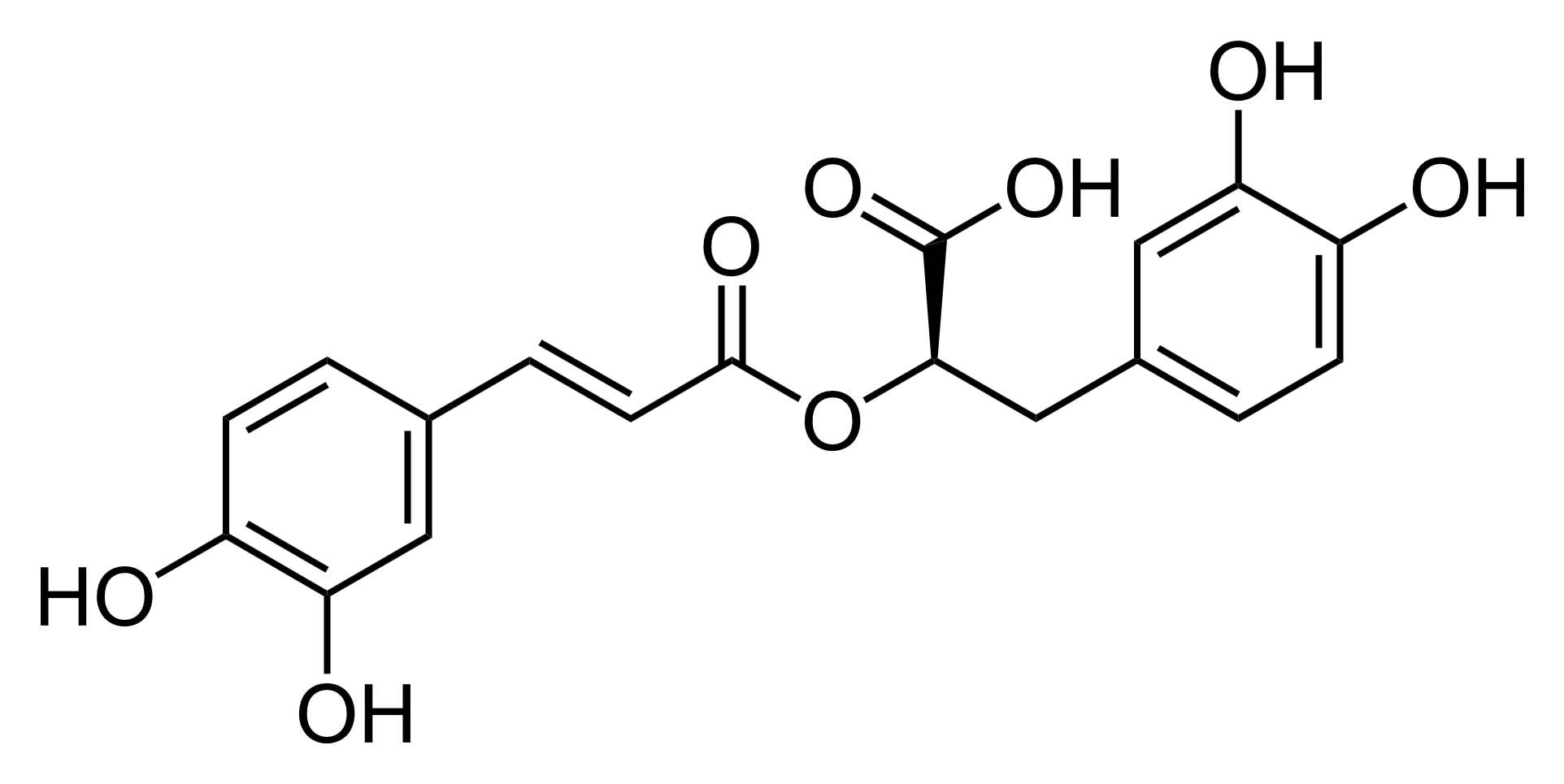
**

………………………………………………………………………………………………………………………………………………………………

**Luteolin 3'-O-β-D-glucuronide (4)**


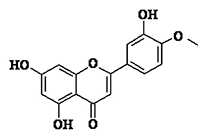


**GlcA**

………………………………………………………………………………………………………………………………………..

**Luteolin (5)**


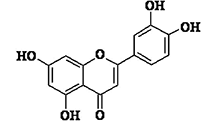


……………………………………………………………………………………………………………………………………………………..

**Apigenin (6)**

**
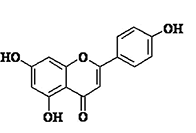
**

………………………………………………………………………………………………………………………………………………………..

**Cirsimaritin(7)**


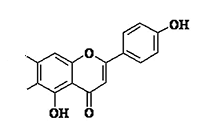


**CH_3_O**

**CH_3_O**

…………………………………………………………………………………………………………………………………………………………..

**Isokaempferide (8)**


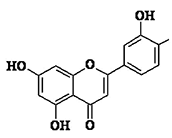


**O CH_3_**

……………………………………………………………………………………………………………………………………………………………

**Penduletin (9)**


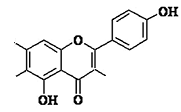


**O CH_3_**

**CH_3_O**

**CH_3_O**

………………………………………………………………………………………………………………………………………………………………

**Xanthomicrol (10)**


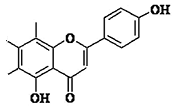


**CH_3_O**

**CH_3_O**

**CH_3_O**

…………………………………………………………………………………………………………………………………………………………………

**Calycopterin (11)**


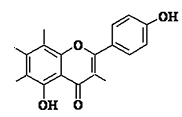


**CH_3_O**

**CH_3_O**

**CH_3_O**

**O CH_3_**
