# Supplementary figures and images for: Overproduction of valuable methoxylated flavones in induced tetraploid plants of Dracocephalum kotschyi Boiss
Source: Bot Stud. 2014 Feb 4;55:22. doi: 10.1186/1999-3110-55-22 (PMC5430325; doi:10.1186/1999-3110-55-22)

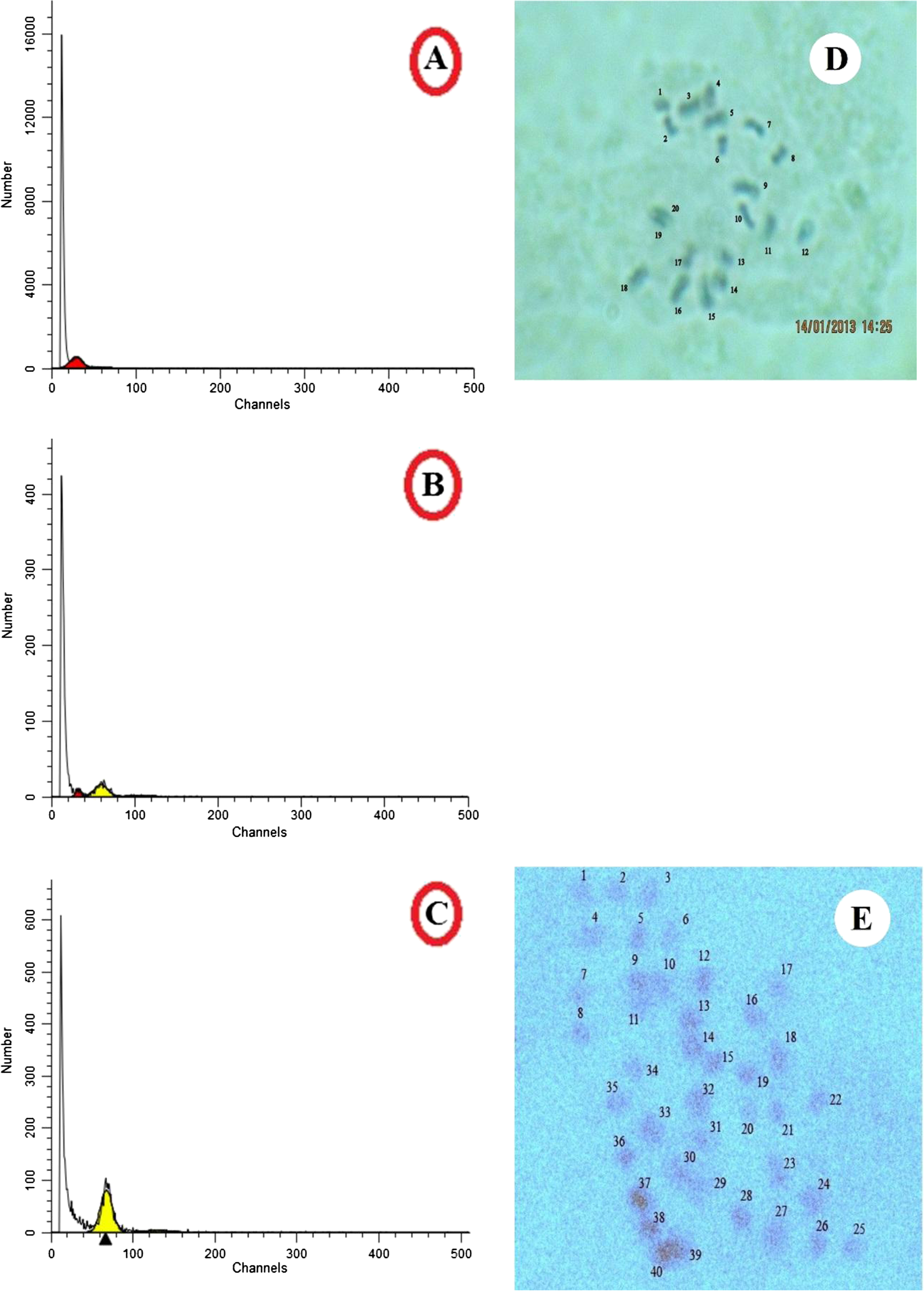

Supplement: Supplementary file 2 — Authors’ original file for figure 1 [file 40529_2013_75_MOESM2_ESM.tif]

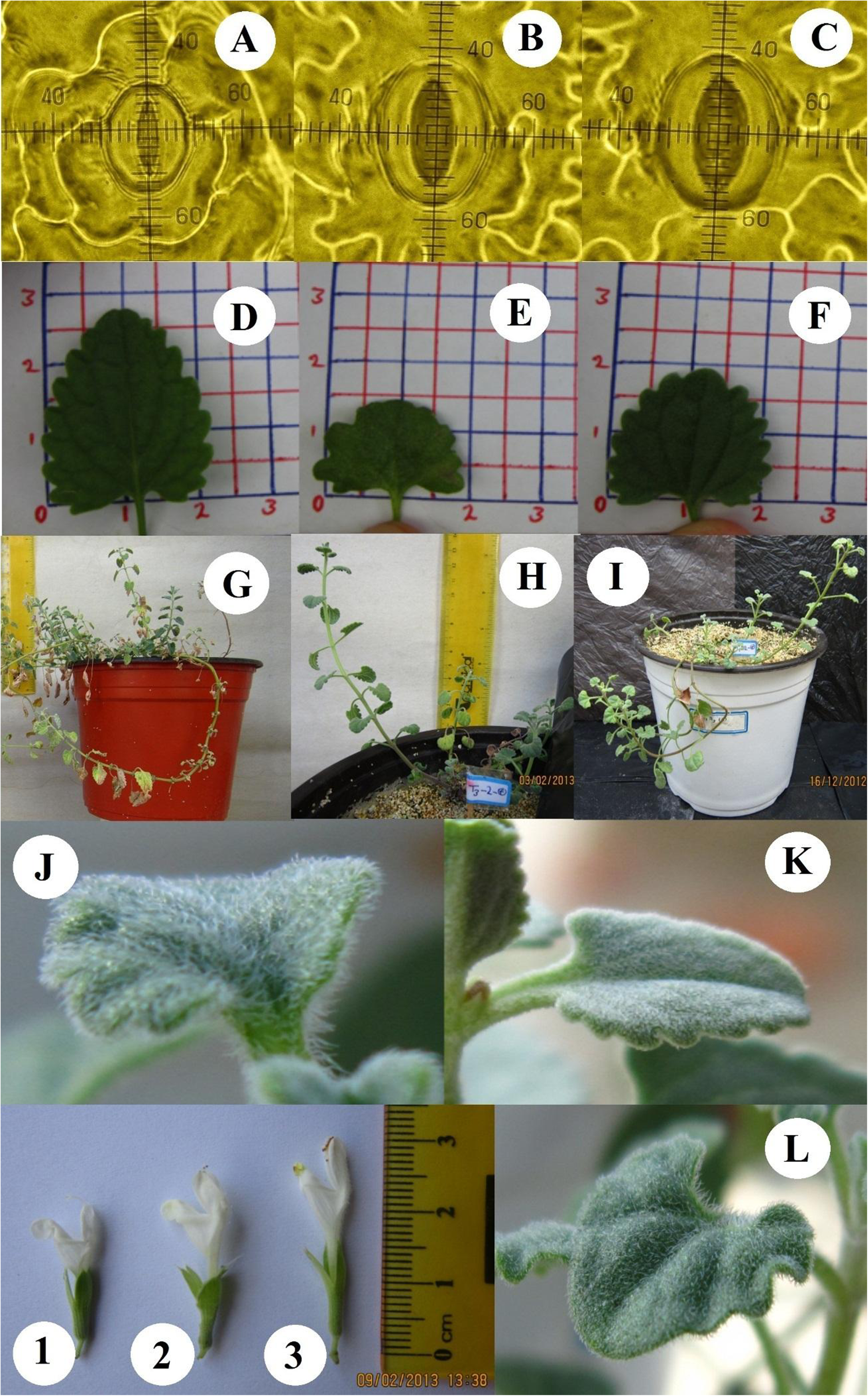

Supplement: Supplementary file 3 — Authors’ original file for figure 2 [file 40529_2013_75_MOESM3_ESM.tif]

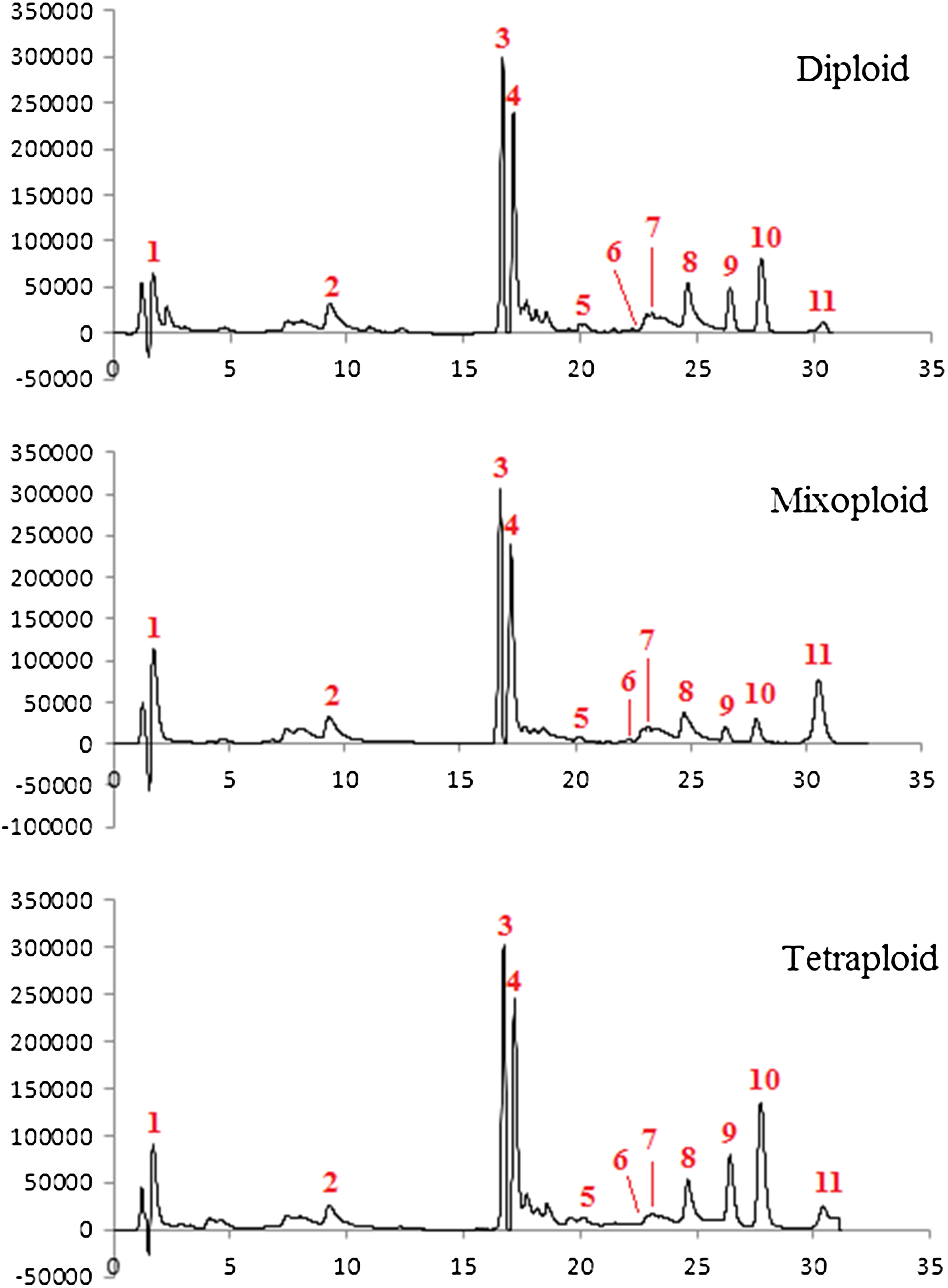

Supplement: Supplementary file 4 — Authors’ original file for figure 3 [file 40529_2013_75_MOESM4_ESM.tif]
